# Who gets frustrated? Identifying individuals prone to frustration using a latent profile analysis

**DOI:** 10.3389/fpsyg.2025.1483965

**Published:** 2025-02-21

**Authors:** Hannaneh Yazdi, Mikael Ljung Aust, Casper Wickman, Aleksandra Bujacz, Leo Kowalski, Johan N. Lundström

**Affiliations:** ^1^Department of Clinical Neuroscience, Karolinska Institutet, Stockholm, Sweden; ^2^Volvo Cars, Gothenburg, Sweden; ^3^Department of Learning, Informatics, Management and Ethics, Karolinska Institutet, Stockholm, Sweden

**Keywords:** frustration, emotions, latent profile analysis, individual differences, emotional regulation

## Abstract

**Introduction:**

Frustration is a complex negative emotion with multifaceted components that significantly influence cognitive and behavioral responses. While previous studies have explored frustration, identifying distinct groups of individuals prone to frustration has yielded inconsistent findings. This study employs a person-centered approach to identify clusters of drivers based on frustration triggers and emotional responses to frustrating events.

**Methods:**

A total of 2,219 drivers participated in an online survey assessing frustration experiences in various frustrating scenarios. Latent Profile Analysis (LPA) was conducted to identify subgroups based on frustration triggers and emotional responses. Predictor variables included Driving Behavior Dimensions (violations, errors, and lapses), user group categories (car-sharing, ownership, leasing), and demographic factors (age and gender).

**Results:**

LPA identified four distinct frustration profiles: Minimal, Low, Moderate, and Severe. These profiles were associated with different frustration triggers (i.e., goal blockage, limited control) and emotional responses (i.e., anger, stress, and irritation). The Severe profile, characterized by a high probability of individuals being highly prone to frustration, exhibited the highest frustration levels and was predominantly composed of older drivers (>45 years), particularly women, from car-sharing and leasing user groups. Emotional response patterns and the likelihood of frustration arousal are consistent across profiles, varying primarily in intensity.

**Discussion:**

These findings offer insights into frustration susceptibility and underscore the need for targeted interventions to enhance emotion regulation in driving contexts. Future research should explore personalized strategies to mitigate frustration based on individual and group characteristics.

## Introduction

Frustration is a complex human emotion (Broom, 1998) that significantly influences cognitive and behavioral processes (Jeon, 2015). Negative emotions such as frustration have been extensively studied in relation to traffic safety as contributing factors to aggressive driving behaviors. Drivers experiencing frustration are prone to demonstrating increased risk-taking and non-compliant behaviors, both of which contribute to heightened accident risk (Jeon, 2015; Bjureberg et al., 2023; Jeon et al., 2014; Shinar, 1998), and aggressive driving is a frequently associated factor in road accidents (Dula and Ballard, 2003; James and Nahl, 2000). Despite these associations, research into the specific triggers and mechanisms underlying frustration in driving contexts remains limited, partly due to the multifaceted nature of frustration and its overlapping dimensions with other negative emotions. Addressing these gaps is essential for developing targeted interventions to reduce aggressive driving and improve traffic safety.

Frustration is a common negative emotional response that arises when individuals encounter obstacles or hindrances while striving to achieve their goals (Dollard et al., 1939; Lazarus, 1994). It encompasses a spectrum of emotional responses, ranging from mild irritation to intense anger, and is influenced by both situational and individual factors (Dollard et al., 1939; Miller, 1941; Berkowitz, 1990). Frustration is also recognized as a multifaceted emotion (Bruun et al., 2016), with numerous factors contributing to its manifestatio, including personal traits (Costa and McCrae, 1999), cognitive appraisals (Lazarus and Folkman, 1984), self-regulation, and expectations (Carver and Scheier, 2001), with numerous factors contributing to its manifestatio. As a first step, it is necessary to classify clusters of factors influencing frustration that are specifically relevant to traffic safety.

Typically, driving is perceived as a straightforward journey to a destination, often idealized in advertisements as a pleasant experience. However, the reality frequently deviates from this ideal. According to Reason et al. (1990), driving is a goal-oriented activity that is essential for daily tasks such as commuting, taking children to school, and shopping, but it is often disrupted by various traffic obstructions. For instance, consider a situation of needing to reach work or attend an important doctor’s appointment. Despite leaving home with adequate time, unforeseen events—such as accidents, road construction, or navigation system malfunctions—can delay arrival. These disruptions create a gap between drivers’ expectations for the journey and their actual experiences, often leading to frustration.

Previous research indicates that frustration during driving is commonly attributed to factors such as traffic jams, congestion, accidents, construction, and delays at red lights during peak hours or slow-moving vehicles on rural roads. Frustration in driving, however, extends beyond such traffic-related issues. For example, Bosch et al. (2020) identified three main categories of driving situations that can lead to frustration: (a) traffic-related situations, such as construction sites, traffic jams, and parking limitations; (b) in-vehicle situations, including difficulties during driving preparation, challenges interfacing with the vehicle, and the social atmosphere within the car; and (c) self-inflicted factors, such as stress, impatience, or distraction; and (d) adverse weather conditions.

Notably, frustration differs from anger, as anger is directed at someone perceived as responsible for an undesirable event (Wirtz and Strohmer, 2017) and varies in both intensity and arousal (Grimm et al., 2007). However, persistent frustration may escalate into anger and lead to aggressive behavior (Shinar, 1998; Berkowitz, 1989). Aggressive driving, in turn, can be a symptom of frustrated (Shinar, 1998).

Furthermore, heightened stress levels are commonly observed in frustrated drivers (Jeon and Zhang, 2013), and frustration has been shown to impair the cognitive skills required for safe driving (Jeon, 2015; Lee, 2010). In the context of driving-related frustration, previous studies have emphasized that frustration can significantly impact both road safety and the user experience (Löcken et al., 2017; Oehl et al., 2019).

Furthermore, frustration can be experienced and expressed in various ways depending on individual differences. Personal traits and coping mechanisms significantly influence how frustration is perceived and managed, in addition to the specific nature of the driving-related events. Gross (2008) emphasized that individuals differ in their emotional, behavioral, and physiological responses, as well as in their subsequent emotional regulation. These differences can also be linked to other emotional responses to frustration (e.g., anger, irritation, disappointment), physiological responses (e.g., increased heart rate, elevated cortisol levels), and behavioral expressions (e.g., aggressive behavior, verbal outbursts, task disengagement).

Understanding the triggers of frustration and identifying driver profiles prone to frustration constitute essential knowledge for developing strategies and interventions to mitigate or prevent frustration in traffic. For example, the link between aberrant driving behaviors and frustration suggests that interventions aimed at curbing such behaviors may reduce frustration. Harris and Nass (2011) conducted a driving simulator study in which frustration was deliberately induced. Their findings indicated that a voice assistant, which reassured drivers by explaining that other drivers’ reactions were not intended to offend, led to improved driving performance and fewer negative emotions.

Similarly, using an empathetic voice assistant that responds to detected anger with messages like, “Hey, are you alright? I can understand that you are a bit angry, sometimes I feel the same way. How about some music to take your mind off things?” has been demonstrated to reduce negative emotions (Braun et al., 2019).

In conclusion, frustration encountered during driving can undermine road safety (Berkowitz, 1989; Lee, 2010). Addressing and reducing frustration is, therefore, crucial. However, the triggers of frustration are diverse and vary among individuals (Bosch et al., 2020; Ferreri and Mayhorn, 2020). Accordingly, this study investigates the multifaceted nature of frustration in various driving situations, employing Latent Profile Analysis (LPA) to explore the interaction between individual characteristics and frustration.

The LPA method is particularly advantageous for identifying and categorizing distinct subgroups within a population based on their responses to frustration. This approach facilitates a nuanced understanding of how individuals experience and react to frustrating scenarios. Our primary objective is to identify the factors that trigger drivers’ frustration and determine which subgroups of drivers are particularly susceptible. We examine three critical dimensions: (1) personality traits, (2) group use categories, and (3) emotional responses to frustrating scenarios. Accordingly, we aim to answer the following Research Questions (RQs):

RQ1. In which specific driving scenarios do drivers most frequently experience frustration?

RQ2. How many distinct driver profiles can be identified based on their frustration levels in various driving situations?

RQ3. How do these driver profiles differ in their emotional responses to frustration?

RQ4. What driver characteristics predict classification into frustration-prone profiles?

We apply a person-centered analytical approach to categorize drivers into distinct profiles based on their responses to various frustration triggers and their associated emotional reactions. By focusing on drivers in Sweden, this study provide a comprehensive understanding of the factors contributing to frustration.

## Method

### Participants

A total of 2,219 drivers in Sweden participated in the study (see participants’ descriptive characteristics in Table 1. They were recruited from three user groups: car-sharing, leasing, and owners. The groups varied slightly in their age distribution. The car-sharing group was younger, with a higher proportion of participants in the 25–34 years (32.5%) and 35–44 years (29.8%) age ranges. In contrast, the car-owner group had most participants in the 45–54 (30.3%) and 55–64 years (26.5%) age groups, while the car-leasing group showed a more mixed age distribution, with most participants in the 35–44 years (23.3%), 55–64 years (38.2%), and 55–64 years (23.3%) age ranges. Additionally, 29 participants were excluded from the dataset as they identified their gender outside the binary option of “Man” and “Woman” for the analysis, since their small number limited the potential for meaningful comparisons.

**Table 1:** Participant descriptive characteristics.

	Total (*N* = 2,219)	Sharing (*n* = 537)	Owner (*n* = 514)	Leasing (*n* = 1,168)
Gender				
Men	1768(80.7%)	349(68.0%)	491(95.9%)	928(79.7%)
Women	422(19.3%)	164(32.0%)	21 (4.1%)	237(20.3%)
Age				
17–24	36 (1.6%)	20 (3.9%)	0 (0.0%)	16 (1.4%)
25–34	341(15.4)	168(32.5%)	15 (2.9%)	158(13.5%)
35–44	516(23.3%)	158(29.8%)	85 (16.6%)	273(23.3%)
45–54	699(31.5%)	99 (18.7%)	155(30.3%)	445(38.2%)
55–64	463(20.9%)	56 (10.3%)	135(26.5%)	272(23.3%)
65–74	133 (6.00%)	19 (3.7%)	110 (21.5%)	4 (0.3%)
74+	17 (0.76%)	5 (1.0%)	12 (2.3%)	0

### Procedure

The data were collected as part of a larger survey examining frustration and flow in driving. The online survey, accompanied by a cover letter, was distributed via email to participants who completed it anonymously. The study adhered to the ethical principles outlined in the Declaration of Helsinki and was reviewed by the Swedish Ethical Review Board (DNR 2020-04337). All participants provided electronic informed consent before participating in the study. Participation was voluntary, and no compensation was offered.

### Measures

The following measures were used in for this study.

#### Frustrating situations measures

Frustration arises when goal-directed actions are impeded (Lazarus and Folkman, 1984) and can be influenced by factors such as motivation or the desire to achieve a specific goal (Amsel, 1992; Dollard et al., 1939), lack of control (Lawrence, 2006), and heightened time pressure (Rendon-Velez et al., 2016). To capture these elements, we incorporated various factors known to induce frustration, such as obstacles to goal-directed behavior and time constraints, to develop frustration-related items for our study. This approach aligns with existing research on frustration and prior studies on driving-related frustration, which suggest that combining these factors effectively induces a sense of frustration in participants (Lee, 2010; Rendon-Velez et al., 2016; Lazarus, 1991).

The frustration scenarios were developed based on a comprehensive literature review (Shinar, 1998; Bosch et al., 2020), internal company reports on challenges in-vehicle use, consultations with experts in road safety, User Experience (UX), and customer satisfaction, as well as discussions with subject matter experts. The questionnaire used in this study was developed to measure reactions to specific frustrating driving scenarios and was previously subjected to Exploratory Factor Analyses (Yazdi et al., 2024). We then grouped the driving situation items into thematic parcels based on shared frustration triggers. Items reflecting similar frustration experiences, such as goal obstruction or road obstructions, were assigned to the same parcel. Expert consultations ensured that these parcels represented meaningful, real-world driving situations, thereby enhancing the construct validity of the frustration measure. The frustration scenarios encompassed influencing factors, including traffic conditions, weather, driver mood, conditions inside and outside the vehicle, and car systems.

Participants were asked to assess the likelihood of different situations leading to frustration. Specifically, they were instructed: “For each of the driving situations, indicate how likely it is that this will contribute to your frustration.” Responses were recorded using a 7-point Likert scale, ranging from 1 (strongly disagree) to 7 (strongly agree), with 4 (neither agree nor disagree) serving as the neutral midpoint. The specific statements corresponding to the frustrating situations are detailed in Table 2.

**TABLE 2:** Parcels of frustrating situations.

Parcel name	Parcel no.	M (SD)	Item no.	Frustrating situation	M (SD)
*Road Obstruction*	1	3.34 (1.85)	2	Driving behind a big truck and not able to have full vision	3.47 (1.87)
			3	Driving in a construction zone	3.15 (1.80)
			4	Being in a traffic jam	4.39 (1.90)
*Attention Requirement*	2	3.73 (1.81)	1	Driving on a road with bicyclists/pedestrians/children	3.22 (1.84)
			5	Driving on a fast-moving highway	4.40 (1.77)
			6	When I am driving in an unfamiliar area and have problems reaching my destination	3.50 (1.77)
			7	Driving in or around a crowded city centre	4.84 (1.87)
*Limited Control*	3	2.83 (1.68)	10	Driving when it rains heavily	2.87 (1.64)
			20	When the car does not function as I expect it to	2.80 (1.71)
*Lacking Resources*	4	3.83 (1.87)	11	Driving in darkness/night	3.78 (1.85)
			19	Being unsure of how to operate the systems in the car (e.g., navigation, pairing your phone)	3.78 (1.87)
			12	Driving when I feel sleepy and tired	3.92 (1.91)
*Goal Achievement Obstructed*	5	5.48 (1.58)	13	When I’m running late to my destination	5.51 (1.56)
			21	When the car does unpredictable things (e.g., when a system does not work)	5.45 (1.59)
*Unpleasant Experiences*	6	5.06 (1.71)	14	Driving when I am not happy about going to my destination (e.g., unpleasant appointment)	4.79 (1.82)
			15	Driving when passengers are noisy	5.33 (1.59)
*Uncomfortable Experiences*	7	4.15 (1.76)	16	Driving when the car is full of passengers	4.31 (1.80)
			17	When the car is dirty	4.68 (1.74)
			18	Hearing loud and annoying noises while driving	3.63 (1.75)
			9	Careless driving by others (e.g., someone changes lanes without indicating)	3.83 (1.80)
*General*	8	3.81 (1.78)	All above +8	Driving when there is a vehicle close behind	2.41 (1.66)

It is important to note that the response scale reflects the probability that a situation will be frustrating rather than the severity of the frustration itself. Nonetheless, the latent profiles identified in the analysis indicate varying degrees of susceptibility to frustration, conceptualized as the likelihood of frustration across different driving scenarios. The 20 items representing various frustrating situations were grouped into eight thematic parcels. However, one of the parcels (Parcel #8: “Driving when there is a vehicle close behind”) was excluded from the final model due to estimation issues, resulting in a final analysis with seven parcels.

Parceling involves combining single items into aggregated parcel, which are then used as indicators of the target latent construct instead of the individual items themselves. The final parcels were categorized as follows (see Table 2): *Road Obstruction* (Parcel #1): Items related to traffic jams, construction zones, and driving behind large vehicles that obstructed the driver’s view. *Attention Requirement* (Parcel #2): Items associated with driving situations that require heightened attention, such as driving near pedestrians or in unfamiliar areas. *Limited Control* (Parcel #3): Items reflecting situations where the driver felt a lack of control, such as when the car was malfunctioning or when weather conditions impaired driving. *Lacking Resources* (Parcel #4): Items representing situations where drivers lacked the necessary resources, such as being unsure of how to operate car systems or driving when tired. *Goal Achievement Obstructed* (Parcel #5): Items reflecting situations where the driver’s goal (e.g., reaching a destination) was blocked or delayed, such as being late for an appointment or encountering unexpected obstacles. *Unpleasant Experiences* (Parcel #6): Items related to non-traffic-related factors that made the driving experience unpleasant, such as passengers being noisy or going to an unpleasant appointment. *Uncomfortable Experiences* (Parcel #7): Items reflect discomfort caused by the driving environment, such as crowded or dirty cars. *General* (Parcel #8): A general indicator that included all items, including one that could not be categorized into any specific parcel (e.g., driving with a vehicle close behind).

#### Behavioral dimension measures

The Driver Behavior Questionnaire (DBQ) section of the survey was also analyzed. This component aimed to measure aberrant driver behaviors using 12 items categorized into three dimensions: *Violations*, *Errors*, and *Lapses*. Participants were asked to rate the frequency of these behaviors as drivers on the road using a 5-point Likert-type scale, ranging from 1 (never) to 5 (always), with intermediate options labeled as 2 (rarely), 3 (sometimes), and 4 (often). The items were derived from a three-dimensional model of the DBQ focusing on *violations*, *lapses*, and *errors* (Lajunen et al., 2004; Martinussen et al., 2013; Rosli et al., 2017).

#### Emotional responses to frustration measures

Participants were asked to describe one of the most frustrating events that had happened to them while driving. They were presented with a list of 15 emotions (*anger, disappointment, sad, stress, anxious, bored, afraid, helpless, tired, stupid, irritated, annoyed, embarrassed*, and *unmotivated*) to select from in response to the question: “What emotions did you feel in the situation you just described?” Responses were recorded using the same 7-point Likert scale as described earlier.

### Data analysis

We used Latent Profile Analysis (LPA) to analyze the multidimensional factors affecting frustration. LPA is a person-centered analytical method designed to identify underlying groups or patterns from observed data (Zhang et al., 2019). The purpose of LPA modeling is to determine the number of profiles that best describe the observed data, enabling the identification of flexible subgroups (Zhang et al., 2019). In LPA, participants are assigned a probability of membership in each cluster based on their level of “prototypical” homogeneity with other participants in the same cluster (Morin et al., 2018). The term “latent” is used because the clusters represent an unobserved classification variable, reflecting unseen subgroups within the data.

Specifically, LPA was employed to classify subcategories of individuals with comparable scores based on different intended indicators (Morin et al., 2018, 2020). We incorporated predictors into the full sample for the final LPA solution using the R3Step approach, a three-step model designed to enhance our understanding of how external variables influence latent class membership. The LPA model was estimated using Maximum Likelihood Robust (MLR) estimation. This estimator is appropriate for models that include continuous and categorical data and provides robust standard errors and chi-square tests. DBQ subfactors, along with the basic demographic information, such as age, gender, and user group categories (owner, sharing, leasing), were used as predictors. Additionally, to explore how factors representing emotional response influence the latent classes, we included covariates in the full sample using the BCH (Bolck, Croon, and Hagenaars) approach (Bolck et al., 2004). The BCH procedure was used to estimate the outcome values across the profiles (Bakk and Vermunt, 2016). The software utilized in this analysis included Mplus (Version 8.4; Muthén and Muthén, 2021) and R: *A Language and Environment for Statistical Computing* (version 3.6) (R Core Team, 2019), using the “psych” package (Revelle, 2021).

## Results

The results are presented sequentially to represent this study’s four primary LPA modeling steps. Prior to discussing the associated results, an explanation of the analytic process at each step is included.

### Step 1. Choosing the number of latent profiles

In the first step of the analysis, we established how many classes would best determined the subpopulations that could be inferred from the data. For the frustrating driving situations, we used eight categories: Road Obstruction (Parcel#1), which contains driving situations where some form of blockage appears; *Attention Requirement* (Parcel#2), where extra attention is required due to an external object, fast movement, or unfamiliarity, *Limited Control* (Parcel#3) when the driving environment or a car function is changes or does not work as expected *Lacking Resources* (Parcel#4), when the driver cannot predict events due to inadequate access to external information or car system; *Goal Achievement* (Parcel#5), where driver’s goal-oriented action is effected and cannot be met, such as failing to reach the desired destination on time, *Obstructed Unpleasant Experiences* (Parcel#6), when the driver is in an unpleasant mood or an external cause triggers the negative feeling, *Uncomfortable Experiences* (Parcel#7), when the external environment creates an uncomfortable state, *General* (Parcel#8) is included all driving situations, including item 8 in the questionnaire, which did not belong to any other described items. This last item specifically pertains to driving with a vehicle closely following behind.

Item 8, “Driving when there is a vehicle close behind,” was excluded from the parcels due to estimation issues. However, this item was included in the “General” indicator, representing an overall mean of frustration in driving across all situations. This indicator was incorporated into the model to better differentiate between shape and level effects (Morin et al., 2017). In total, eight parcels were used in the LPA analyses.

The parcel *Goal Achievement Obstructed* (*M* = 5.477, *SD* = 2.033) elicits the highest level of frustration across the whole sample. Similarly, the parcel *Unpleasant Experiences* also showed a high overall level of frustration (*M* = 5.059, *SD* = 2.297). Conversely, *Limited Control* (*M* = 2.834, *SD* = 1.833) displayed the most lowest overall impact on frustration likelihood. *Road Obstruction* situations elicited a moderate level of frustration (*M* = 3.67, *SD* = 2.521). These results reveal that frustrating situations associated with *Goal Achievement Obstructed* and *Unpleasant Experiences* generally provoke a higher likelihood of frustration across profiles than *Limited Control* and *Road Obstruction*.

**Figure 1:**
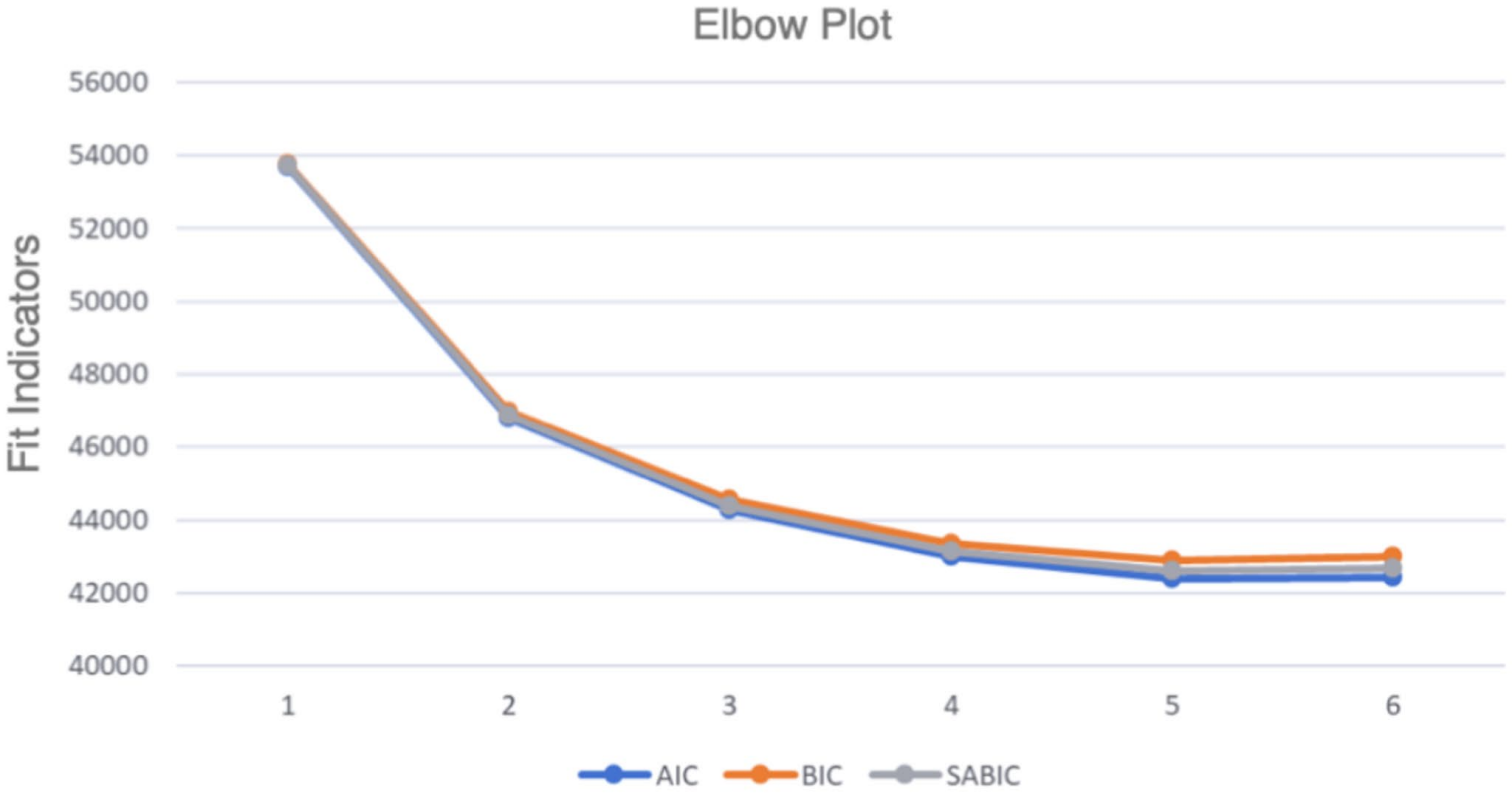
The elbow plot of model classes with fit indicators (AIC, BIC, SABIC).

Models with one to six classes were compared. Several fit indicators were used to assess the quality of the models (see Table 3 and Figure 1). Log-Likelihood (LL), which measures how well the model fits the data, indicates a better fit with higher (less negative) values; Akaike Information Criterion (AIC), evaluating model complexity, with lower values indicating a better trade-off between model fit and complexity, Bayesian Information Criterion (BIC), penalizes model complexity more strongly than AIC, with lower values suggesting a better model fit considering complexity. Sample-Size Adjusted BIC (SABIC) is similar to BIC but adjusted for sample size, with lower values indicating a better fit. *Entropy* reflects the clarity of class separation, with values closer to 1 indicating better separation between classes. While the model fit indices showed slight improvements with the five-class solution (and subsequently with six classes), we selected the four-class solution based on several considerations. First, the four-class model balanced statistical fit and interpretability, aligning with the principle of parsimony. Second, the additional class in the five-class solution represented a small subgroup that overlapped substantially with existing profiles, offering limited new insights.

**TABLE 3:** Fit indicators for latent class analysis.

Classes	LL	AIC	BIC	SABIC	Entropy
1	−26,825	53,682	53,771	53,720	–
2	−23,366	46,798	46,981	46,876	0.911
3	−22,086	44,273	44,550	44,391	0.895
4	−21,425	42,985	43,357	43,144	0.897
5	−21,117	42,403	42,869	42,602	0.912
6	−21,117	42,437	42,998	42,677	0.921

LL, Log-Likelihood; AIC, Akaike Information Criterion; BIC, Bayesian Information Criterion; SABIC, Sample-Size Adjusted Bayesian Information Criterion; Entropy, A measure of classification accuracy.

To determine the appropriate number of latent profiles, we compared models with one to six classes using several fit indices, including the Akaike Information Criterion (AIC), Bayesian Information Criterion (BIC), and Sample-Size Adjusted BIC (SABIC). The entropy value was also examined to assess the clarity of profile classification. The four-profile solution was selected as it provided a balance between model fit and parsimony, with AIC = 42,985.386, BIC = 43,357.456, SABIC = 43,144.597, and entropy = 0.897. While models with more profiles (e.g., five or six profiles) showed slightly better fit indices, they introduced redundancy and reduced interpretability. Therefore, the four-profile solution was retained for subsequent analyses.

We labeled the four profiles based on frustration likelihood: *Minimal*, *Low*, *Moderate*, and *Severe*. These labels represent relative differences in the probability of experiencing frustration across driving situations. Profile 1 (Minimal) represents 12% of the population and includes individuals who generally experience low frustration across driving situations. Profile 2 (Low), which accounts for 37% of the population, is characterized by modest frustration. Profile 3 (Moderate), representing 40% of the population, indicates significant frustration, though not at extreme levels. Profile 4 (Severe), comprising 11% of the population, exhibits the highest frustration levels across all indicators. The final solution was based on both empirical indicators (i.e., model fit and model improvement) as well as the theoretical meaningfulness of the profiles included in the analyses.

**Figure 2:**
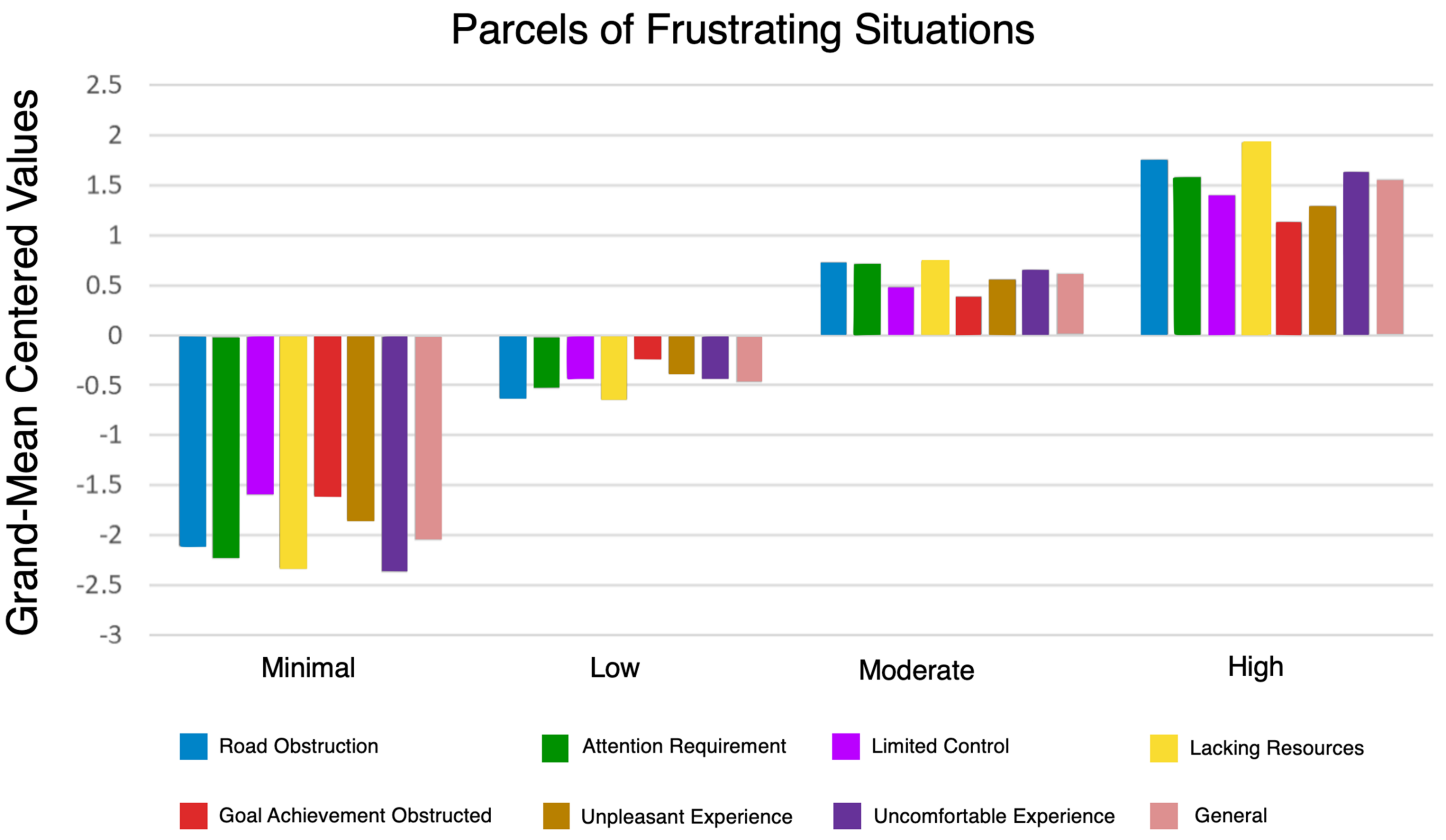
The final four profiles are classified based on their experience of frustration associated with each parcel. Profile indicators are represented as factor scores with grand-mean centered values for each respective class. Values above zero represent scores higher than the sample mean; values below zero represent scores lower than the sample mean.

Figure 2 provides an overview of the grand-mean-centered values of the four-profile solution indicators associated with frustrating situations clustered into eight parcels. The variances of indicators were allowed to vary within each profile. A summary of the means and variances is presented in Table 4. The four profiles primarily differ in level rather than shape, with all indicators, including the general one, following the same gradient from low to high frustration across different driving situations. The *Severe* profile consistently scores above the grand-mean-centered values across all frustration indicators, while the *Minimal* profile scores consistently below the mean.

**Table 4:** Summarizes the mean (M) and standard deviation (SD) of each frustrating situation parcel across the four profiles.

Class	Total		Minimal		Low		Moderate		Severe	
	M	SD	M	SD	M	SD	M	SD	M	SD
*Road Obstruction*	3.670	2.521	1.557	0.510	3.036	1.542	4.400	1.191	5.422	1.287
*Attention Requirement*	3.989	1.957	1.760	0.578	3.463	1.012	4.705	0.585	5.569	0.590
*Limited Control*	2.834	1.833	1.246	0.249	2.396	1.200	3.320	1.164	4.236	1.668
*Lacking Resources*	3.826	2.323	1.500	0.431	3.191	1.240	4.570	0.748	5.750	0.647
*Goal achievement Obstructed*	5.477	2.033	3.872	4.582	5.244	1.674	5.859	0.702	6.609	0.244
*Unpleasant Experiences*	5.059	2.297	3.209	3.654	4.671	1.901	5.614	0.762	6.353	0.431
*Uncomfortable Experiences*	4.113	1.892	1.747	0.619	3.682	0.964	4.770	0.417	5.745	0.429
*General*	4.002	1.213	1.958	0.309	3.526	0.200	4.622	0.094	5.559	0.207

According to Figure 2, the *Minimal* profile scores consistently fall below the grand-mean-centered values for all indicators. The *Low* profile scores are around the mid-range of the sample mean, indicating moderate frustration levels in various frustrating situations. The *Moderate* profile indicates high frustration, with scores above the sample mean but not the highest. The *Severe* profile scores above the sample mean in all driving situations, indicating severe frustration.

The Latent Profile Analysis in Table 4 presents the likelihood of frustration levels across various frustrating situations for each profile, revealing distinct patterns. Across the four latent profiles, the total Mean and Standard Deviation (SD) of frustration for each frustration situation were analyzed to understand overall trends.

### Step 2. Investigating emotional responses to frustration

**Figure 3:**
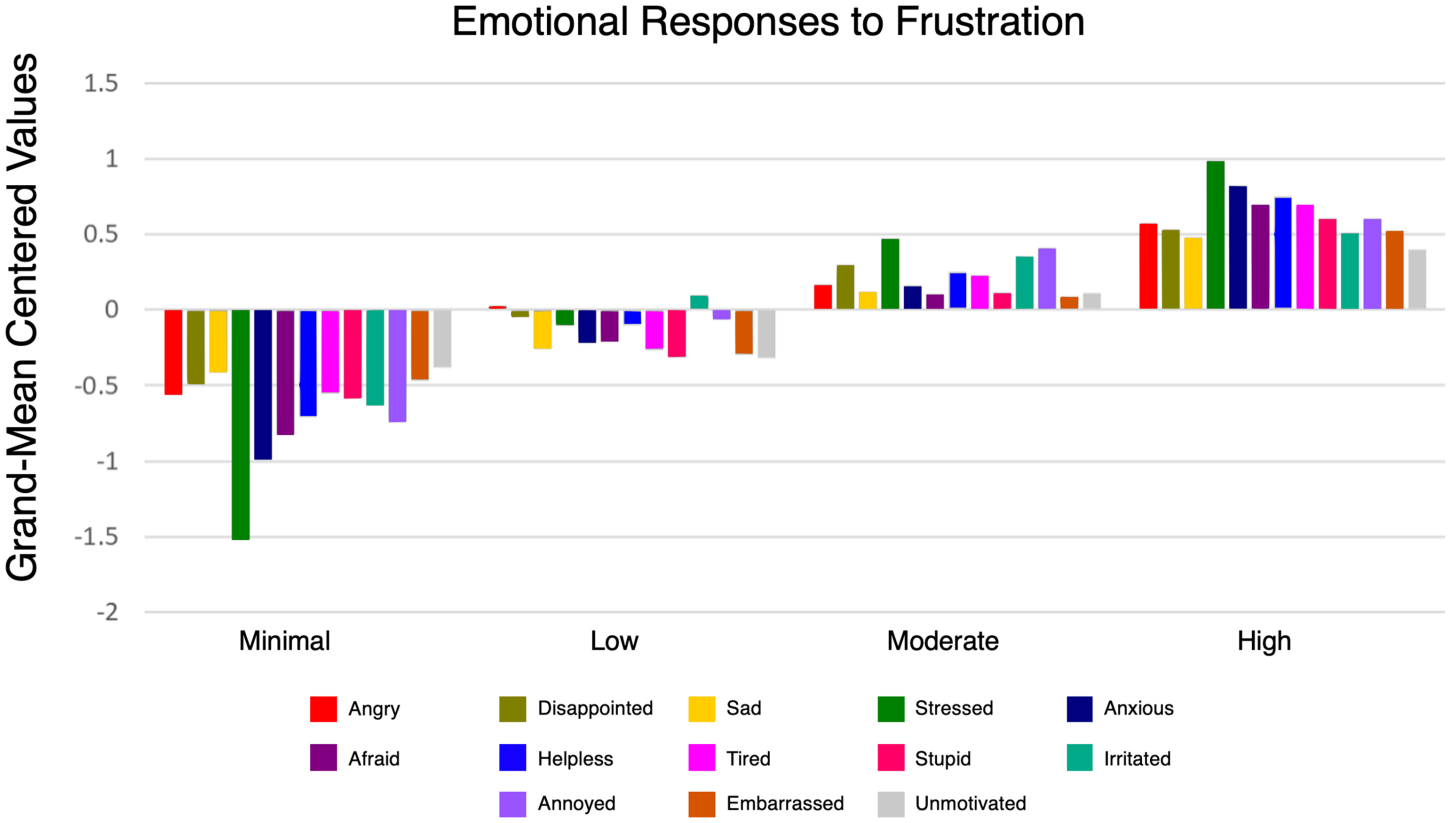
Overview of frustration differences across four latent profiles using grand-mean centered values. Values above zero represent scores higher than the sample mean; values below zero represent scores lower than the sample mean.

In the second step of the analysis, outcomes variables were added to the chosen model to validate and further explore the meaning of the latent profiles. We included indicators of 14 emotional states from the emotional response to frustration scale. Figure 3 provides an overview of differences in how members of the four latent profiles experience and respond to frustration. The item “Bored” was excluded from the figure because it exhibited minimal variation across profiles. The differences in mean values for all outcome variables are presented in Table 5.

**TABLE 5:** Profile membership and emotional responses of frustration.

	Minimal	Low	Moderate	Severe
Angry	4.445	5.036 ^a^	5.171 ^a^	5.577
Disappointed	3.651	4.092	4.435 ^a^	4.668 ^a^
Sad	2.656 ^a^	2.810 a	3.184	3.542
Stressed	2.934	4.351	4.920	5.436
Anxious	2.602	3.370	3.794	4.411
Bored	2.920 ^a^	2.732 ^a^	2.833 ^a^	2.972 ^a^
Afraid	2.204	2.819	3.135	3.728
Helpless	2.655	3.264	3.614	4.116
Tired	2.313	2.602	3.086	3.554
Stupid	1.729	2.007	2.426	2.916
Irritated	4.818	5.542	5.800 ^a^	5.954 ^a^
Annoyed	3.906	4.586	5.051 ^a^	5.249 ^a^
Embarrassed	1.604 ^a^	1.778 ^a^	2.155	2.593
Unmotivated	1.959 ^a^	2.025 ^a^	2.453 ^b^	2.741 ^b^

Values marked with the same superscript letter are similar within each row. All other values are significantly different from one another (*p* < 0.05).

The differences in Means are presented.

As illustrated in Figure 3 and detailed statistically in Table 5 significant differences in the emotional states experienced across the four profiles. The *Minimal* profile showed relatively lower scores on emotional states such as *Angry* (M = 4.445), *Stressed* (M = 2.934), and *Anxious* (M = 2.602) compared to higher frustration profiles. Conversely, the *Severe* Profile exhibited the highest scores for *Angry* (M = 5.577), *Stressed* (M = 5.436), and *Anxious* (M = 4.411), indicating a more intense emotional response. Additionally, the *Severe* profile reported higher levels of *Irritated* (M = 5.954) and *Annoyed* (M = 5.249) compared to other profiles. Emotional responses such as *Embarrassed* and *Unmotivated* were relatively low across profiles but still showed notable variation, with the *Minimal* profile having the lowest scores for *Embarrassed* (M = 1.604) and *Unmotivated* (M = 1.959). These findings highlight how frustration manifests differently across profiles, with higher frustration profiles consistently reporting more intense negative emotions.

### Step 3. Adding predictors

In the final step of the analysis, predictors were added to the model. These included demographic variables (age and gender) and drivers’ characteristics (user group categories and DBQ behavioral dimension). Table 6 presents the results of categorical latent variable multinominal logistic regressions for the predictors in the model.

**TABLE 6:** Predictors of profile membership.

	Minimal profile		Low profile		Moderate profile	
	logit	OR	logit	OR	logit	OR
Age > 45	−0.184	0.832*	−0.173*	0.841*	−0.038	0.963
Women	−0.471	0.624*	−1.052***	0.349***	−0.526**	0.591***
Sharing	−3.746***	0.024***	−1.996***	0.136***	0.239	1.270
Leasing	−2.323***	0.098***	−1.076***	0.341***	0.621	1.860
DBQ violations	−0.129	0.879	0.340	1.404	−0.073	0.929
DBQ errors	−0.291	0.747	−0.185	0.831	0.127	1.136
DBQ lapses	−1.626***	0.197***	−0.557**	0.573***	−0.192	0.825

This table highlights significance with asterisks for logit and OR values, indicating statistically significant relationships. *p-values* correspond to logit coefficients from the regression model. In this table, “logit” represents the natural logarithm of the odds ratio for membership in a specific profile relative to the Severe profile. “OR” (Odds Ratio) is the exponentiated value of the logit, providing an interpretable measure of how the odds change with a one-unit increase in the predictor. Significance is determined based on the logit coefficient, and the *p-value* corresponds to this coefficient. Since the OR is derived from the logit, it shares the same significance level, which is marked with asterisks in the table.

Severe profile was selected as the reference. * < 0.05; ** < 0.01; *** < 0.001.

Predictors of membership in profiles were compared with the Severe profile as the reference group. Table 7 provides a concise overview of these profiles — *Minimal*, *Low*, *Moderate*, and *Severe* —including frustration likelihood, demographic trends, key characteristics, driving behavior dimensions, and associated emotional responses. All interpretations are based on comparisons of membership relative to the Severe profile, which serves as the reference.

**Table 7:** Comprehensive characterization of individuals belonging to each frustration-prone subgroup.

Profile	Frustration likelihood	Demographics	Key characteristic	Driving behavior dimensions	Emotional responses
Minimal	Modest	*Comprises 12% of the sample* Older individuals (>45 years) are modestly less likely to belong. Women are significantly less likely to belong.	Strongly negative association with car-sharing behaviors. Significantly lower lapses compared to the Severe profile.	Significantly lower likelihood of lapses.	*Low arousal*: minimal irritation, anger, and annoyance. Low in embarrassed, stupid, and unmotivated
Low	Temperate	*Comprises 37% of the sample* Older individuals (>45 years) are slightly less likely to belong to the Low profile. Women are significantly less likely to belong to the Low profile.	Individuals in car-sharing and leasing user groups are significantly very unlikely to belong to Low profile.	Significantly lower likelihood of lapses.	*Moderate arousal*: mild irritated, angry, and annoyed. Low in embarrassed, stupid, and unmotivated
Moderate	High	*Comprises 40% of the sample* No significant relationship with individuals’ age. Women are significantly less likely to belong to this profile.	No significant relationship	No significant relationship.	*High arousal*: notably irritated, angry, and annoyed. Low in embarrassed, stupid, and unmotivated
Severe	Very High	*Comprises 11% of the sample* Higher likelihood of older individuals (<45 years). Women are significantly less likely to belong to the Minimal, Low, or Moderate profiles relative to the Severe profile.	Severe profile members are more likely to engage in sharing and leasing user groups compared to Minimal or Low profile but similar to Moderate.	Severe profile members are significantly more likely to exhibit lapses compared to Minimal and Low profiles.	*Very high arousal*: extremely irritated, angry, and annoyed. Low in embarrassed, stupid, and unmotivated

The profiles range from Severe (highly prone to frustration) to Minimal (low-prone).

#### The *Minimal* profile

Compared to the *Severe* Profile (reference group), being over 45 years was not significantly associated with the likelihood of membership in the Minimal Profile (logit = −0.184, OR = 0.832, *p* > 0.05). Women were significantly less likely to belong to this profile compared to the *Severe* Profile, as indicated by a significant association (logit = −0.471, OR = 0.624, *p* < 0.05). Car-sharing and leasing user groups were strongly negatively associated with *Minimal* profile membership, with sharing user groups showing a significant reduction in likelihood (logit = −3.746, OR = 0.024, *p* < 0.001) and leasing user group similarly showing a strong negative association (logit = −2.323, OR = 0.098, *p* < 0.001). These results highlight how demographic and behavioral factors differentiate profiles. Additionally, DBQ lapses were significantly associated with a decreased likelihood of being in the *Minimal* Profile (logit = −1.626, OR = 0.197, *p* < 0.001), suggesting that individuals in this profile are less prone to driving lapses. In contrast, DBQ violations (logit = −0.129, OR = 0.879, *p* > 0.05) and errors (logit = −0.291, OR = 0.747, *p* > 0.05) did not significantly predict membership in the *Minimal* Profile.

#### The *Low* profile

For the *Low* Profile compared to the *Severe* Profile, being over the age of 45 years was significantly associated with a reduced likelihood of membership (logit = −0.173, OR = 0.841, *p* < 0.05). Women participants had a significantly lower probability of belonging to the *Low* Profile compared to the Severe profile (logit = −1.052, OR = 0.349, *p* < 0.001). A high level of sharing user group membership was strongly negatively associated with membership in the *Low* Profile (logit = −1.996, OR = 0.136, *p* < 0.001). Similarly, the leasing user group was significantly associated with a reduced likelihood of being in the *Low* Profile (logit = −1.076, OR = 0.341, *p* < 0.001). DBQ violations did not present any significant prediction of membership in the *Low* profile (logit = 0.340, OR = 1.404, *p* > 0.05). DBQ errors also did not significantly predict membership in the *Low* Profile (logit = −0.185, OR = 0.831, *p* > 0.05). However, DBQ lapses were significantly associated with a reduced likelihood of membership in the Low Profile (logit = −0.557, OR = 0.573, *p* < 0.01), suggesting that individuals in this profile are less prone to driving behavioral of lapses than individuals in the Severe profile.

#### The *Moderate* profile

In comparison to the *Severe* Profile, being over 45 years old was not significantly associated with membership in the *Moderate* Profile (logit = −0.038, OR = 0.963, *p* > 0.05). Women were significantly less likely to belong to the *Moderate* Profile compared to the *Severe* Profile (logit = −0.526, OR = 0.591, *p* < 0.01). Neither sharing user group membership (logit = 0.239, OR = 1.270, *p* > 0.05) nor leasing user group (logit = 0.621, OR = 1.860, *p* > 0.05) were statistically significant predictors of membership in the *Moderate* Profile. Similarly, neither DBQ violations (logit = −0.073, OR = 0.929, *p* > 0.05) nor DBQ errors (0.127, *OR* = 1.136, *p* > 0.05) significantly predicted membership in this Moderate profile. Additionally, DBQ *lapses* also did not significantly predict membership in the *Moderate* profile (*logit* = −0.192, *OR* = 0.825, *p* > 0.05).

#### Predictors of membership in the Severe profile

The *Severe* Profile served as the reference group in the Latent Profile Analysis, allowing for comparisons across membership predictors in other profiles. This profile consists of individuals over 45 years old who were generally less likely to belong to the *Minimal* Profile (*logit* = −0.184, *OR* = 0.832, *p* < 0.05) and the *Low* Profile (*logit* = −0.173, *OR* = 0.84, *p* < 0.05). Women were significantly less likely to be in *the Minimal* and *Low* profiles compared to *the Severe* profile, suggesting that women have a higher likelihood of being in *Severe* profile. The high level of sharing user group membership was strongly negatively associated with the *Minimal* (*logit* = −3.746, *OR* = 0.024, *p* < 0.001) and *Low* profiles (*logit* = −1.996, *OR* = 0.136, *p* < 0.001) but not with the *Moderate* profile, suggesting that individuals in the *Severe* profile engage more in sharing user group. Similarly, a high level of leasing user group membership was significantly associated with a decreased likelihood of being in the *Minimal* profile (*logit* = −2.323, *OR* = 0.098, *p* < 0.001) and *Low* profile (*logit* = −1.076, *OR* = 0.341, *p* < 0.001), but not the *Moderate* profile, predicting that there are more individual belonging to leasing user group associated with *Severe* profile. DBQ *lapses* was significantly negatively associated with the *Minimal* profile (*logit* = −1.626, *OR* = 0.197, *p* < 0.001) and Low profile (*logit* = −0.557, *OR* = 0.573, *p* < 0.01) but not with the *Moderate* profile, suggesting higher levels of DBQ *lapses* in the *Severe* profile. DBQ violations and errors did not significantly differentiate the profiles, implying a similar distribution of these behaviors across all profiles. Thus, the *Severe* profile is characterized by older age, particularly women, higher engagement in sharing and leasing behaviors, and higher levels of DBQ lapses.

## Discussion

The primary objective of this study was to identify and analyze latent profiles of drivers experiencing varying levels of frustration across different driving situations. Through Latent Profile Analysis (LPA), we identified four distinct profiles of frustration: *Minimal*, *Low*, *Moderate*, and *Severe*, representing different likelihood of frustration. The *Severe* profile, which comprises 11% of the sample, demonstrated the highest frustration levels across all driving situations, while the *Minimal* profile, representing 12%, showed consistently low frustration levels. Individuals in the Severe Profile were more likely to be older drivers (>45 years), more likely to be women, engaged in shared driving services, and highly associated with DBQ lapses. However, we observed similar patterns in both frustration situations and emotional responses to frustration across the four identified profiles, although with varying intensity. These findings support the idea that triggers and responses to frustration can be similar. However, differences in frustration experiences among individuals within each cluster may be mediated by demographic and behavioral factors. In this study, these factors included age, gender, behavioral dimensions, and user group categories, which emerged as key determinants of variations in frustration intensity across the profiles.

All identified profiles reveal both similar patterns and distinct levels of emotional responses in drivers’ frustration experiences across profiles. Boredom and unmotivated responses exhibited minimal variation across profiles, maintaining consistent intensity. However, individuals in the *Severe* profile experienced significantly higher levels of high-arousal emotional responses to frustration, with higher mean scores than other profiles. Conversely, the *Minimal* Profile exhibited resilience, with lower mean scores across all emotional responses. This supports the notion that higher likelihood of frustration arousal is linked to more intense emotional responses (Lazarus and Folkman, 1984). Interestingly, Shorkey and Crocker (1981) note that individuals respond to frustration in either adaptive or maladaptive ways. Adaptive responses are constructive, aiming to resolve the issue preventing goal achievement — either by avoiding the problem beforehand or employing problem — solving strategies once it arises. Based on the higher frustration levels observed in the *Severe* Profile, it is plausible to propose that the predominantly older drivers in this group may exhibit maladaptive responses to frustrating events. This pattern is consistent with theories of emotion regulation and coping. Managing emotions by controlling environmental triggers is a lifelong strategy that becomes more self-directed with age. As individuals gain control over their surroundings and emotional challenges, their ability to regulate emotions improves, especially in older adults (Thompson, 1991; Charles et al., 2001; Charles and Piazza, 2009). This finding is in contrast with our observation as the older drivers are more likely to be in the Severe profile, suggesting they are less able to manage frustrating situations.

Frustration levels were highest in scenarios involving *Goal Achievement Obstructed* (e.g., running late or encountering unexpected obstacles) and *Unpleasant Experiences* (e.g., noisy passengers or unpleasant appointments). Conversely, *Limited Control* (e.g., malfunctioning car systems or poor weather) and *Road Obstruction* (e.g., traffic jams) were associated with a significantly lower likelihood of frustration. Additionally, demographic variables and the DBQ *lapses* dimension were significant predictors of profile membership.

The higher likelihood of experiencing frustration in situations triggered by Goal Achievement Obstructed, such as “Driving behind a big truck and not being able to have full vision,” “Driving in a construction zone,” and “Being in a traffic jam,” aligns with previous studies indicating that the importance of the goal, along with the intensity of the desire to achieve it, have a strong connection to frustration (Dollard et al., 1939; Franz et al., 2020; Gross et al., 1997).

A closer examination of unpleasant experiences, such as “Driving when I am not happy about going to my destination (i.e., unpleasant appointment)” and “Driving when passengers are noisy,” suggests that baseline mood or other underlying factors may be associated with experiencing frustration. This finding is consistent with the idea that non-traffic-related factors, such as emotional state and interpersonal stress, play a role in driving frustration, especially for those in the Severe Profile. In these frustrating situations, neither the unpleasant appointment nor the noisy passengers directly induces frustration in the driver, as they are not related to the traffic situation or car interface, and we can consider them as the moderator factors in increasing the likelihood of frustration in drivers. Moreover, the lower level of association between Limited Control and Road Obstruction and experiencing frustration is consistent with theoretical models that emphasize frustration directly to a lack of control and decreased arousal (Donnerstein and Wilson, 1976) and identify traffic situations that are recognized as primary triggers for frustration in driving.

Additionally, car-sharing and leasing behaviors were positively associated with the *Severe* profile, suggesting that individuals engaging in these practices may experience a higher likelihood of frustration levels. This finding suggests that drivers who lease or share cars may have less tolerance for frustration, whereas the car owner user group is less strongly associated with a higher likelihood of frustration levels. While drivers who own their cars have a lower likelihood of experiencing frustration, this finding might support the notion of a direct association between the experience of frustration and contrast with expected outcomes (Lazarus, 1994), as the sharing group, which likely consists of more naive and less experienced drivers, might exhibit a lower tolerance for unexpected experiences, resulting in less resilience to frustrating situations. This suggests that helping drivers in the *Severe* profile develop strategies to handle unexpected outcomes more effectively could be a useful approach to minimizing frustration in traffic.

As for the DBQ dimensions, while prior studies suggest that violations and errors have the most pronounced impact on aggressive driving behavior and traffic accidents (Özkan and Lajunen, 2005), our findings indicate that *Violations* and *Errors* did not significantly differentiate the profiles. However, DBQ *lapses* was significantly lower in the Minimal and Low Profiles compared to the Severe Profile, suggesting that lapses in driving behavior are more strongly associated with higher frustration levels. *Lapses*, on the other hand, were significantly lower in the *Minimal* and *Low* profiles compared to the *Severe* profile. This finding suggests that DBQ lapses is the most important driver behavioral dimension to focus on when trying to prevent frustration. Lapses require dedicated attention in future research to better understand their role in frustration regulation.

This study contributes to applied social psychology by elucidating the complex interplay between driving situations, emotional responses to frustration, and individual differences in experiencing frustration. Identifying distinct frustration profiles provides a deeper understanding of how different drivers experience and react to frustration and can inform more tailored interventions. These insights can inform the development of targeted interventions to mitigate frustration and enhance driving experiences. For instance, tailored stress management programs could be designed for high-frustration profiles, while promoting car-sharing initiatives might alleviate frustration for specific demographic groups. Furthermore, the findings underscore the importance of considering demographic and behavioral factors in understanding driver frustration. Policymakers and transportation planners can leverage this knowledge to create more inclusive and supportive driving environments. For example, designing road infrastructures that minimize goal obstruction and unpleasant experiences could reduce frustration and improve traffic flow.

This study was conducted as an exploratory analysis of cross-sectional data, which introduces several limitations. One is that it was conducted using self-reported data, which can introduce biases such as social desirability bias, recall bias, and subjective interpretation of questions. Participants might underreport or overreport their experiences and emotions related to driving frustration as the experience might be close to the self-report time. We recommend that future studies focus on a longitudinal approach over time and use real-time data collection with a combination of objective measures, such as physiological stress indicators, to complement self-reported data and provide a more comprehensive understanding of driver frustration. Moreover, while the study categorizes various driving situations, it does not encompass all possible scenarios drivers can encounter. Hence, other significant driving situations that influence frustration levels might not have been included in the current analysis, potentially overlooking critical aspects of the driving experience. Also, while the LPA approach allowed for identifying distinct profiles, further research is needed to explore the underlying mechanisms that drive the profile differences and explore whether similar profiles can be found in other populations. Therefore, we proposed to include other potential predictors, such as socioeconomic status, personality traits, and cultural factors, that were not considered here in future studies. We chose to apply a person-centered approach, Latent Profile Analysis (LPA), to identify distinct driver profiles based on their frustration levels because this approach allows for a clearer understanding of how individual characteristics and driving experiences may create subgroups with unique patterns of frustration. While LPA offers valuable insights into how distinct subgroups experience frustration, we recognize the merit of comparing this approach to a variable-centered approach such as factor analysis, which might provide a more global understanding of frustration as a continuous factor. As suggested by Kam and Zhou (2016), comparing these two approaches could provide useful insights into the relative fit of each model and their ability to explain relationships with external variables. We acknowledge that conducting a factor analysis on the frustration scale would allow us to assess its psychometric properties and potentially enhance the validity of the subgroups identified in our study. While parcels served as an appropriate data reduction technique for conducting LPA in this study, their use assumes a formative measurement model. This assumption requires further investigation to validate the instrument’s structure and ensure replicability in future studies.

In conclusion, this study comprehensively analyzes individual differences frustration through LPA, providing a comprehensive analysis of drivers’ frustration and identifying subgroups more prone to experiencing it. This work opens new avenues for future studies to move toward a more personalized and human-centric approach to developing coping strategies and interventions for road safety.

## Data Availability

The raw data supporting the conclusions of this article will be made available by the authors, without undue reservation.
